# Outcomes of cochlear implantation in Usher syndrome: a systematic review

**DOI:** 10.1007/s00405-023-08304-2

**Published:** 2023-11-06

**Authors:** Hannah Louisa Cornwall, Chon Meng Lam, Daoud Chaudhry, Jameel Muzaffar, Peter Monksfield, Manohar L. Bance

**Affiliations:** 1 Cardiff and Vale University Health Board, Cardiff, UK; 2 College of Medical and Dental Sciences, University of Birmingham, Birmingham, UK; 3 Department of Clinical Neurosciences, University of Cambridge, Level 3, A BlockCambridge Biomedical Campus, Box 165, Cambridge, CB2 0QQ UK; 4 Department of Otolaryngology, University Hospitals Birmingham NHS Foundation Trust, Birmingham, UK; 5 Department of Otolaryngology, Addenbrooke’s Hospital, Cambridge University Hospitals NHS Foundation Trust, Cambridge, UK

**Keywords:** Usher syndrome, Cochlear implant, Hearing loss, Quality of life

## Abstract

**Purpose:**

This study is a systematic review of the literature which seeks to evaluate auditory and quality of life (QOL) outcomes of cochlear implantation in patients with Usher syndrome.

**Methods:**

Systematic review of studies indexed in Medline via PubMed, Ovid EMBASE, Web of Science, CENTRAL and clinicaltrials.gov was performed up to March 9th 2022, conducted in accordance with the PRISMA statement. Patient demographics, comorbidity, details of cochlear implantation, auditory, and QOL outcomes were extracted and summarized.

**Results:**

33 studies reported over 217 cochlear implants in 187 patients with Usher syndrome, comprising subtypes 1 (56 patients), 2 (9 patients), 3 (23 patients), and not specified (99 patients). Auditory outcomes included improved sound detection, speech perception, and speech intelligibility. QOL outcomes were reported for 75 patients, with benefit reported in the majority.

**Conclusions:**

Many patients with Usher syndrome develop improved auditory outcomes after cochlear implantation with early implantation being an important factor.

**Supplementary Information:**

The online version contains supplementary material available at 10.1007/s00405-023-08304-2.

## Introduction

Usher syndrome, also known as Hallgren syndrome, Usher-Hallgren syndrome, retinitis pigmentosa-dysacusis syndrome, and dystrophia retinae dysacusis syndrome, is an autosomal recessive hereditary ciliopathy characterized by partial or complete sensorineural hearing loss and progressive visual loss due to retinitis pigmentosa [1]. It affects 3–6 persons per 100,000, and in the United States accounts for approximately 50% of all deaf-blindness [2].

Mutations in at least 10 genes thought to account for Usher syndrome are present in both inner ear hair cells and retinal photoreceptors, where they lead to loss of hair cells in the cochlea and progressive loss of rod and cone photoreceptors in the retina [1]. Most patients retain central vision of around 20/40 until age 40 [1]. Peripheral visual losses impair the use of sign language to mitigate communication challenges in this population. Notably, auditory nerve function, gross anatomy and intellectual ability are not typically affected, making these patients good candidates for cochlear implantation.

It is important that patients and their families can make an informed decision based on the best available evidence on cochlear implantation outcomes. Usher syndrome affects several aspects of a patient’s life, many of which cannot be accurately assessed by audiometric tests alone. For instance, the impact of Usher syndrome on a patient’s schooling needs to be considered when clinicians counsel patients and their families about cochlear implants and this knowledge can provide useful insight for teachers educating patients with Usher syndrome also. The use of validated Patient Reported Outcome Measures (PROMs) in measuring cochlear implant outcomes provides further insight into the effects of Usher syndrome on a patient’s quality of life and can also be a useful tool for assessing treatment effectiveness. To our knowledge, there are no systematic reviews in the literature at present that assesses the audiometric outcomes, schooling and PROMs for cochlear implants in patients with Usher syndrome.

Three clinical subtypes of Usher syndrome are recognized, distinguished by severity of hearing loss, age of onset of symptoms, and vestibular involvement (Online Resource 1).

Type 1 is characterized by pre-lingual hearing loss and early onset visual loss and early cochlear implantation in this group has been associated with better auditory outcomes in children with severe or profound congenital hearing loss [3].

Pre-lingual hearing loss in type 2 is mild to severe with preservation of low-frequency hearing, often amenable to amplification by acoustic hearing aids [4].

Type 3 is the least common, accounting for 4% of cases [1]. Progressive sensorineural hearing loss and visual loss develop post-lingually in late childhood, adolescence, or adulthood. Acoustic hearing aids and, more uncommonly, cochlear implants are treatment options in this population, but optimal timing is unclear.

Vestibular function may sometimes be negatively affected by cochlear implantation. However, there is very little evidence of implant-associated vestibular deterioration in Usher syndrome [5].

### Objective

In this systematic review, we provide an overview of auditory and PROMs after cochlear implantation in patients with a confirmed diagnosis of Usher syndrome. We aim to provide clinicians with a synthesis of evidence with which to counsel patients and their families on the range of outcomes a patient with Usher syndrome may experience following cochlear implantation.

## Methods

### Study identification and selection

We conducted a systematic literature search using the databases PubMed, Ovid EMBASE, ISI Web of Science, the Cochrane Register of Controlled Trials and ClinicalTrials.gov from inception to March 9th 2022 using the MeSH and key search terms: cochlear implant*, electric acoustic stimulation, hearing aid, Usher syndrome and derivatives (Online Resource 2). Search results were manually deduplicated. The review was conducted in accordance with the PRISMA statement.

Abstracts were screened for relevance by two independent reviewers (HLC and CML). Two reviewers (two of HLC, CML, DC) independently evaluated full texts for inclusion using predetermined eligibility criteria. Bibliographies of included studies were searched for additional relevant studies. Discrepancies were resolved through consensus or consultation with a third reviewer.

### Inclusion and exclusion criteria

PICO eligibility criteria were used:

- Population: adults or children with a confirmed diagnosis of Usher syndrome.
- Intervention: cochlear implant, unilateral or bilateral.
- Comparison: any or none.
- Outcome: post-implantation (1) objective measurements of sound detection, speech recognition, speech intelligibility, and/or (2) PROM scores including quality of life measures.

Exclusion criteria: (1) animal study, (2) pharmacological model, (3) genetic diagnosis of Usher syndrome without associated phenotype, (4) mixed groups where data from patients with Usher syndrome cannot be extracted, (5) opinion, editorial or review article, (6) non-English language, (7) full text unavailable.

Studies from the same institution were assessed using treatment dates and authorship to determine the likelihood of overlapping datasets and those deemed to be at high risk are grouped in this review.

### Data extraction and synthesis

Two reviewers independently extracted data from included studies using standardized Microsoft Excel (Redmond, WA) spreadsheets piloted during our preliminary literature search, that were compared to ensure accuracy. Inconsistencies were resolved through consensus. Mean and range data for patient age, outcome data and time to last follow-up were recorded by preference where available. Where figure resolution permitted accurate extraction, graphical data were used.

### Quality assessment

Two reviewers independently scored included studies for evidence quality using the OCEBM 2011 Levels of Evidence 2.1 grading system [6]. Each study was assessed using the 2012 risk of bias checklist for quality assessment of non-randomized studies (adapted for case series where required) [7].

The protocol for this systematic review was registered prospectively in the PROSPERO database and can be found at https://www.crd.york.ac.uk/PROSPERO/display_record.php?RecordID=185102.

## Results

**Fig. 1:**
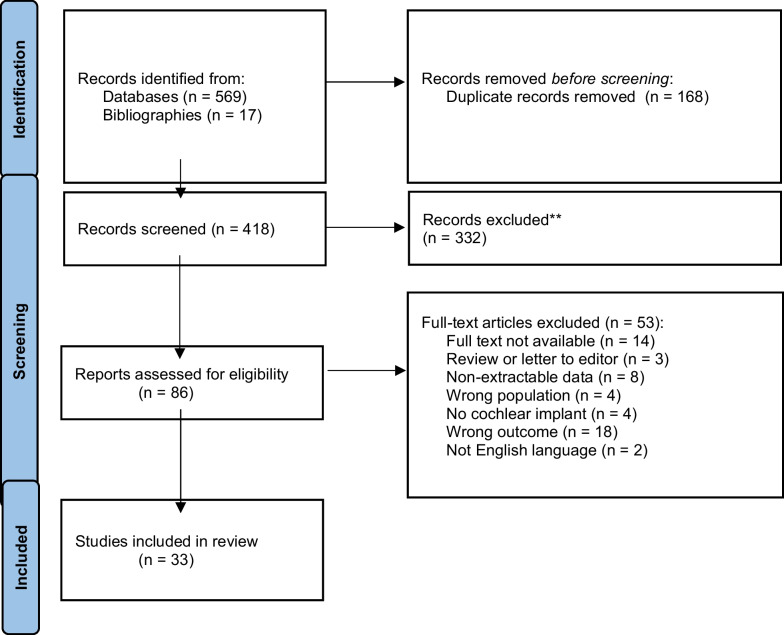
Preferred reporting items for systematic reviews and meta-analyses flow diagram. From: Page MJ, McKenzie JE, Bossuyt PM, Boutron I, Hoffmann TC, Mulrow CD, et al. The PRISMA 2020 statement: an updated guideline for reporting systematic reviews. BMJ 2021;372:n71. https://doi.org/10.1136/bmj.n71. For more information, visit: http://www.prisma-statement.org/

Thirty-three publications met our inclusion and exclusion criteria after full-text screening (Fig. 1), representing the experiences of 187 patients with Usher syndrome and cochlear implant(s). Diagnoses included Usher type 1 (56 patients), Usher type 2 (9 patients), Usher type 3 (23 patients), and Usher of non-specified type (99 patients). 120 unilateral and 29 bilateral CI insertions were reported; five studies did not specify whether CI insertion was unilateral or bilateral (Table 1).

**Table 1:** Study demographics

References	Country	Patients (implants)	Insertion (patients)	M:F	Visual acuity (patients)	Intellectual impairment (patients)	Anatomic abnormal-ity (patients)	Mean age at first implant in months (range)^b^	Mean age at second implant in months (range)^b^	Implant used (implants)	Complications
*Usher type: 1*											
Hinderink[18]/Vermeulen [25]^a^	The Nether-lands	4 (5)	U (4)	2:2	Impaired	N.S	N.S	254.4 (161–347)	278	3M/Vienna (1), MedEl E/1 (1), Nucleus 22 (3)	Implant failure and reimplant (1)
Saeed [28]	UK	1 (ns)	N.S	0:1	Impaired	N.S	N.S	Child < 60	–	Nucleus 22M	N.S
El-Kashlan [27]	USA	1 (1)	U (1)	1:0	N.S	N.S	N.S	42	–	Nucleus 22 (1)	Implant failure at 3y and reimplant
Loundon [29]	France	11 (11)	U (11)	N.S	Impaired (7)	Behavioral disorder (1)	None	81.9 (19–240)	–	Nucleus 22 or 24 (10), Clarion HF (1)	N.S
Pennings[30]/Damen[33]^a^	The Nether-lands	14 (15)	U (15)	N.S	Impaired (11)	N.S	N.S	149^c^ (42–365)	N.S	Nucleus 22 (8) or 24 (7)	Implant failure (2) and reimplant (1), facial nerve stimulation (1)
Liu [16]	USA/UK	9 (9)	U (9)	N.S	N.S	N.S	N.S	65 (24–132)	–	Nucleus 22 (3), 24M (5), or Clarion 1.2 (1)	N.S
Henricson [40]	Sweden	7 (13)	U (1), Bsim (6)	N.S	N.S	None	N.S	26 (9.5–48)	58 (18–144)	N.S	N.S
Imtiaz [19]	Saudi Arabia	3 (ns)	N.S	1:2	N.S	None	None	37 (16–72)	–	Nucleus 22 (1), 24C (2)	N.S
Hoshino [22]	Brazil	10 (10)	U (10)	7:3	Impaired (10)	N.S	N.S	227 (60–588)	–	Nucleus 24M/K (5), freedom (2), Digisonic SP (1), MedEl Sonata (1), AB HiRes 90k (1)	N.S
*Usher type: 2*											
Ruiz and Gomez 2013[15]	Colombia	1 (2)	Bseq (1)	0:1	Blind	N.S	Partial ossification left cochlea	400	409	Nucleus freedom (1), AB Harmony (1)	None
Hartel [20]	The Nether-lands	8 (8)	U (8)	1:7	N.S	N.S	N.S	708 (564–876)	–	Nucleus multichannel (6), AB HiRes 90 k (2)	N.S
*Usher type: 3*											
Loundon [29]	France	1 (1)	U (1)	N.S	Impaired	None	None	528	–	Nucleus 22 or 24 (1)	N.S
Pietola [21]	Finland	19 (19)	U (1)	7:12	Impaired	N.S	None	492 (120–768)	–	Nucleus multichannel (12), MedEl multichannel (7)	N.S
Wahlqvist [11]	Sweden	3 (3)	U (1)	0:3	N.S	N.S	N.S	N.S	–	N.S	N.S
*Usher type: unclassified*											
Dawson [26]	Australia	3 (3)	U (1)	N.S	Blind (3)	None	N.S	219 (179–241)	–	Nucleus 22 (3)	Electrode deactivation due to short-circuit (1)
Chute and Nevins [36]	USA	3 (3)	U (1)	1:2	Impaired (1), blind (2)	N.S	N.S	102 (78–114)	–	Nucleus 22 (most)	N.S
Jenison [17]	USA	2 (2)	U (1)	1:1	Impaired (2)	Learning and behaviour problems (1)	None	Child	–	Nucleus 22 (2)	None
Shiomi [31]	Japan	1 (1)	U (1)	0:1	N.S	N.S	None	Adult	–	Nucleus 22	N.S
Waltzman [37]	USA	1 (1)	U (1)	N.S	N.S	None	N.S	53	–	Nucleus 22 or 24 or Clarion	None
El-Kashlan [27]	USA	1 (1)	U (1)	0:1	Blind	N.S	N.S	384	–	Clarion (1)	None
Derinsu and Ciprut [8]	Turkey	1 (1)	U (1)	0:1	N.S	N.S	N.S	624	–	Nucleus 24 M	N.S
Loundon [29]	France	1 (1)	U (1)	N.S	Impaired	None	None	36	–	Nucleus 22 or 24 (1)	N.S
Gifford and Revit [34]	USA	1 (1)	U (1)	N.S	N.S	N.S	N.S	191	–	AB HiRes 90 k	N.S
Withers [9]	Australia	1 (2)	Bseq (1)	0:1	Blind	N.S	N.S	756	804	Nucleus 24R (1), Med-El Sonata (1)	N.S
Serrador-García [14]	Spain	1 (1)	U (1)	0:1	Blind	N.S	N.S	Adult	–	N.S	‘cabaret music’ on disconnecting HA with Charles-Bonnet syndrome
Vincent [38]	France	2 (4)	Bseq (1), Bsim (1)	N.S	N.S	N.S	N.S	43 (30–56)	64 (56–71)	Digisonic SP (4)	N.S
Broomfield [23]	UK	9 (N.S.)	N.S	N.S	N.S	Mild cognitive delay (1)	None	73 (15–216)	–	N.S	None
Janeschik [10]	Germany	7 (13)	U (1), Bseq (6)	N.S	Blind (4)	Cognitive deficit (3)	N.S	51.5 (N.S.)	N.S	Cochlear © devices (13)	N.S
Jatana [35]	USA	26 (38)	U (14), B (12)	17:9	N.S	Pervasive developmental disorder (2)	Hydroceph-alus and microprem (1)	40 (6–139)	61 (N.S.)	N.S	N.S
Alsanosi [13]	Saudi Arabia	1 (2)	Bsim (1)	1:0	N.S	N.S	None	5	5	Med-El Concerto (2)	Significant intraoperative bone marrow bleeding
Alzhrani [12]	Saudi Arabia	9 (N.S.)	N.S	N.S	N.S	None (IQ > 80)	N.S	Child	–	N.S	Reimplant (1)
Mesnildrey [32]	France/UK	1 (1)	U (1)	N.S	N.S	N.S	N.S	84	–	AB HiRes 90 k	None
Nair [24]	India	27 (N.S.)	N.S	18:9	N.S	N.S	None	35 (11–56)	–	N.S	None
Lyutenski [39]	Germany	1 (2)	U (2)	0:1	N.S	N.S	None	456	492	AB HiRes Ultra with Mid-scala electrode array (1), MED-EL Synchrony 2 with FLEX straight lateral wall electrode array (1)	Scalar translocation; implant failure at 3 years and reimplant

‘Impaired’ denotes reported night-blindness or visual field restriction. ‘Blind’ includes legally blind with preserved central vision < 10 degrees and severe visual impairment

*HA* hearing aid; *U* unilateral insertion; *B* bilateral insertion—unclear whether simultaneous or sequential; *Bsim* bilateral simultaneous insertion; *Bseq* bilateral sequential insertion

^a^Pennings 2006 and Damen 2006, likely include patients reported in Hinderink 1994 and Vermeulen 1994

N.S., not specified, ^b^where age not available, adult, child or mixed population given ^c^median

Twenty-seven studies were published in otolaryngology journals, four in ophthalmology journals, and two in general medical journals. Included studies represent diverse contributions from around the world. Studies were conducted in the USA (seven), The Netherlands (five), UK (four), France (three), Saudi Arabia (three), Australia (two), Sweden (two), Brazil (one), Colombia (one), Finland (one), Germany (two), India (one), Japan (one), Spain (one), and Turkey (one). The majority (63%) were retrospective single-center case reports, case series or cohort studies. There were no randomized studies, as might be expected for severe to profound deafness. Four studies from The Netherlands were deemed likely to report from an overlapping dataset and are therefore reported together in this systematic review.

### Auditory outcomes

#### Sound detection

Audiometry data was reported by 16 studies, four pre-implantation [8–11], four post-implantation [12–15], and eight both pre- and post-implantation [8, 16–22] (Online Resource 3). Where reported, pre-operative PTA4 (pure tone average 0.5–4 kHz) was above 90 dB HL across all studies, and all patients exhibited improved sound detection post-operatively.

Five studies [16–19, 22] of Usher type 1 representing twenty-eight patients reported both pre- and post-implant auditory thresholds. Mean pre-implant PTA4 was 113 dB HL. Mean post-implant aided PTA4 was 37.3 dB HL (range 10–50 dB HL).

One study [20] of Usher type 2 representing eight patients reported a mean pre-implant PTA4 of 98 dB HL, and mean post-implant aided PTA4 of 34 dB SPL.

One study [21] of Usher type 3 representing nineteen patients reported a mean pre-implant PTA4 of 110 dB HL, and mean post-implant aided PTA4 of 34 dB HL.

#### Speech perception

Speech perception data was reported by 30 studies [8–10, 12, 13, 15–39], using a wide variety of assessment tools (Online Resource 3). Most patients demonstrated improved speech perception, either closed or open set recognition. At least 19 patients achieved no demonstrable improvement in speech perception at follow-up: all were diagnosed with Usher type 1 or were pre-lingually deaf [22, 23, 29, 30, 35], two had comorbid cognitive conditions unrelated to Usher syndrome [35], and one originated from a country that did not speak the language spoken by rehabilitation center staff [29]. Of those without additional comorbidities or language barriers, eight were adults [22, 30], four were adolescents [22, 30], and four were children implanted after the age of 5 years [22, 23, 30]. Four patients became non-users of their device [22, 23]. Hinderink et al. [18] found no statistically significant differences in cochlear implant outcomes between Usher type 1 and non-syndromic pre-lingually deaf patients—the sample size of the Usher (four patients) and non-Usher group (five patients) in this study was small and the cause of deafness in the non-Usher group was limited to three patients with meningitis, one with Mondini dysplasia and one patient with a hereditary cause.

All patients with Usher type 2 for whom speech perception was reported demonstrated open-set speech recognition post-implantation [15, 20], with seven patients demonstrating improved accuracy in best-aided conditions [20]. In a single study, there was no significant difference in speech perception between patients with Usher type 2a and non-visually impaired patients with post-lingual hereditary deafness [20].

Both studies (representing 20 patients) that reported speech perception post-implantation in patients with Usher type 3 reported significant improvement in accuracy of open-set speech perception[21, 29].

#### Speech production

Speech intelligibility was reported by seven studies [9, 12, 13, 24, 27, 29, 36], four of which reported data as per a classification system: Central Institute for the Deaf Speech Intelligibility Evaluation (CID-SPINE) [36], Speech Intelligibility Rating (SIR) [12, 24], or categories of speech production [29] (Online Resource 3).

Two studies [29, 36] reported pre- and post-implantation speech intelligibility. Improvement was reported in 10 of 12 children (83%) without complex sentence production pre-implantation. From spared words (1 child) or no production (9 children), 50% could produce complex sentences at 9–96 months follow-up [29]. No patients experienced a decline in speech intelligibility post-implantation.

Post-implantation SIR scores were reported to improve with time, from 1.06 at 3 months, to 4.3 at 12 months follow up (mean value for 27 children implanted before 6 years old) [24]. Alzhrani et al. [12] reported a mean SIR score of 4.9 (range 4–5) for 9 children (unspecified duration of follow-up).

#### Schooling

Three studies [17, 18, 22] reported pre-implantation schooling environments for children with Usher type 1: school for the deaf or special school (10 children) [18–22], mainstream school with interpreter (2 children) [17], or mainstream school (interpreter use not specified, 4 children) [22] (Online Resource 3).

Post-implantation, Imtiaz et al. [19] reported that all three of the children with Usher type 1 in their study were in mainstream education, and one of the four patients previously enrolled in a school for the deaf reported by Hinderink et al. [18] was enrolled in mainstream school, sometimes requiring the help of an interpreter (Online Resource 3).

Of those children with Usher type not specified who were of formal education age at follow-up, reported post-implantation schooling included: mainstream school (6 children) [12, 37, 38], mainstream school with sign-language support (1 child) [36], hearing-impaired unit in mainstream school (3 children) [12], school for the deaf (1 child) [36] (Online Resource 3).

In all other studies, educational setting was not reported.

#### Communication mode

Communication preferences were reported in three studies pre-implantation [18, 26, 27], five studies post-implantation [12, 35, 37, 40], and four studies both pre- and post-implantation [8, 16, 22, 31] (Online Resource 3).

Among patients with Usher type 1, pre-implantation communication preferences included oral (5 patients) [16, 22], combination oral/sign (2 patients) [22], sign (9 patients) [16, 22], total or lip-reading (6 patients) [16, 18], and no language (1 patient) [22]. Post-implantation, communication preferences included auditory-oral (9 patients) [16, 22, 40], combination oral/sign (5 patients) [22, 40], sign (5 patients) [22], total or lip-reading (7 patients) [16]. Of the two studies reporting both pre- and post-implant data, Liu et al. [16] report uptake of total amongst the children who used sign pre-implantation, and Hoshino et al. [22] report uptake of supplementary sign in two children who underwent late implantation: one oral child and one child without language.

Pre- but not post-implantation communication preference is reported for four pre-lingually deaf patients with Usher of non-specified type: two used cueing supplement [26], one used sign [27], and one used total [26].

Post- but not pre-implantation communication preference is reported for 44 pre-lingually deaf children and one child with progressive hearing loss with Usher of non-specified type: 30 used auditory-oral [12, 23, 35, 37], two used combination oral/sign [23], two used sign [23], two used a primarily oral form of total [35], two used a primarily manual form of total [35], and two used augmentative communication [35].

Two adult post-lingually deafened patients with Usher of non-specified type communicated via palm writing with [8] or without [31] Braille alphabet pre-implantation, and by auditory-oral communication at post-implant follow-up, including telephone use in one case [8].

Four patients became non-users of their device (7% of USH1, 2% of total population sampled) [22, 23].

No data regarding communication preference was available for patients with Usher types 2 and 3.

### Electroacoustic stimulation

Two patients with Usher syndrome type 2a reported the use of hearing aids for electro-acoustic stimulation in the ear that received a cochlear implant [20]. The authors reported no statistically significant difference in speech perception in cochlear implant-only vs. hearing aid-assisted states using the NVA open speech recognition test.

### PROMs and QOL measures

Seven studies [11, 18, 20, 21, 24, 30, 33] reported PROMs or quality of life (QOL) measures collected via formalized surveys, comprising responses from 75 patients post-implantation: 18 with Usher type 1 [18, 30, 33], 8 with Usher type 2a [20], 22 with Usher type 3 [11, 21], and 27 children with Usher type not specified [24] (Table 2). Survey types included the Gestel-Nijmegen Implant Questionnaire [18], Glasgow Benefit Inventory or Glasgow Children’s Benefit Inventory [20, 21, 24, 30], Nijmegen Cochlear Implant Questionnaire [20, 33], Usher Lifestyle Survey [20, 33], 12-Item Short Form Survey [33], Glasgow Health Status Inventory [21], Health of Equal Terms [13], Hospital Anxiety and Depression Scale [11], and Health Utility Index [24].

**Table 2:** Post-implantation QOL outcomes

References	Usher patients (implants)	Usher type	Outcomes	Mean follow-up in months (range)
Hinderink [18]	4 (5)	1	Gestel–Nijmegen implant questionnaire (number of patients reporting this outcome): Could hear and recognise environmental sounds (4) Felt less isolated (2), more secure or safer (4), more independent or confident (3), more optimistic about the future (1), improved security negotiating traffic (1), enjoyed music (3) Improved interpersonal communication (4) Disappointed in their communication ability (1)—this individual reported that they were often reliant on writing or the help of a familiar person to communicate with unknown people	12 (12)
Pennings [30]/Damen [33]	14 (15)	1	G(C)BI score [−100 to + 100] as mean (range): < 10 years old = 42.3 (+ 20 to + 68) 10–19 years old = 23.3 (0 to + 45) > 19 years old = 5 (−22 to + 25) NCIQ (7 adults, 7 children) [0–100] mean score (range): Sound perception basic: 47.4 (5–85) [adult], 75.4 (52.5–85) [child] Sound perception advanced: 48.3 (27.8–80) [adult], 67.9 (32.5–95) [child] Speech production: 25.4 (12.5–43.5) [adult], 42.5 (22.5–57.5) [child] Self-esteem: 70 (37.5–90) [adult], 65.4 (40–90) [child] Activity limitations: 76 (32.5–90) [adult], 74.2 (63.9–88.9) [child] Social interactions: 65 (37.5–80.6) [adult], 70.9 (60–77.5) [child] Usher Lifestyle Survey: General trend that CI users maintain independence more easily than non-implanted patients, particularly in the domains of 'communication' and 'mobility' CI users, particularly children, tend to report needing more equipment to detect emergency situations at home, or are afraid that they will not notice at night SF12 (7 adult patients only): No significant difference (at *P* < 0.05) when compared to adult USH1 patients without CI	60 (24–120)/109 (36–188)
Hartel [20]	8 (8)	2a	GBI score [−100 to + 100] as mean (SD): Total: 41.6 (10.1) General domain sub-score: 52.0 (15.6) NCIQ [0–00] mean score (range): Sound perception basic: 71 (50–82) Sound perception advanced: 67.6 (50–86.7) Speech production: 88 (78–100) Self-esteem: 69.4 (35.6–84) Activity limitations: 67.8 (54–84) Social interactions: 67.7 (54–88) Usher Lifestyle survey: Most patients used additional equipment to wake up, 4/8 patients used equipment to hear someone at the front door, 6/8 needed help from others to fill out a form, 4/8 used accessory equipment to receive emergency information. 4/8 used a telephone without help, 5/8 used equipment to help them write or read. 6/8 patients required help from relatives or friends to buy food or communicate with a doctor, 5/8 needed help to travel to the shop, 7/8 needed help to travel to the doctor	52.5 (12–228)
Pietola [21]	19 (19)	3	GBI score [−100 to + 100] as mean (SD): Total: 30 (19) Social domain sub-score: 14 (18) Physical domain sub-score: 0 (17) GHSI score [0–100] as mean (SD): Hearing loss-related: 59 (9) Vision-related: 56 (14)	72 (12–156)
Wahlqvist [11]	3 (3)	3	HET and HADS: Self-assessed health: Good (all 3 patients) Poor physical health days (in last 30 days): 3, 4, 0 Poor mental health days (in last 30 days): 3, 2, 0 Days in which poor physical or mental health affected capacity for work and ADLs (in last 30 days): 3, 2, 0 Physical health score (HET): 0, 1, 4 Mental health score (HET and HADS): 1, 2, 1 Social trust score (HET): 0, 1, 1 Total problems reported: 1, 2, 6	N.S
Nair [24]	27 (N.S.)	N.S	GBI mean score: 9/18 HUI version 3.0 mean score: 17/40	12 (12)

*NCIQ* Nijmegen Cochlear Implant Questionnaire; *GCBI* Glasgow Children’s Benefit Inventory; *GBI* Glasgow Benefit Inventory adult format; *SF12* 12-Item Short Form Survey; *GHSI* Glasgow Health Status Inventory; *HET* Health on Equal Terms; *HADS* Hospital Anxiety and Depression Score; *HUI* Health Utility Index; *N.S.* not stated

All studies using relevant measures reported benefits in hearing-related QOL (HR-QOL) and independence. On general (health and social) QOL surveys such as the Glasgow Benefit Inventory, responses were more varied. General QOL negatively correlated with age of implantation and speech perception among patients with Usher type 1, with two patients reporting no improvement or detriment to their QOL [30]. Compared to non-implanted adult patients with Usher type 1, statistically significant benefits in the HR-QOL domains of sound perception and speech production were noted—the former with greater benefit if implanted in childhood, the latter only among patients implanted in childhood [33]. It must be noted that the sample size in this study was very small. Comparing children implanted aged 1–6 years with and without Usher syndrome at 1-year follow-up, Nair et al. [24] noted that HR-QOL increased to a lesser extent among children with Usher syndrome, a result the authors assigned to the presence of multiple sensory handicaps.

Amongst patients with Usher type 2a and Usher type 3, general QOL was consistently improved following implantation [20, 21] and reported mental health and social trust problems in three patients with Usher type 3 were fewer than reported by twelve Usher type 3 patients without cochlear implants [11]. Amongst patients with Usher type 3, no correlation was identified between Glasgow Benefit Inventory score and patient age, age of implantation, or speech or sound perception threshold [21].

### Complications

Complications were reported in eight patients, including significant intraoperative bleeding in the youngest child, implanted age 5 months [13], facial nerve stimulation [30], electrode deactivation due to short-circuit[26], implant failure [18, 27, 30, 33], reimplantation [12, 18, 27, 33, 39], and auditory hallucinations in the context of Charles–Bonnet syndrome [14] (Table 1).

### Rehabilitation

Fourteen studies reported some form of structured auditory rehabilitation program [8, 12, 16–20, 23–29], of which four were integrated with an ongoing educational program or schooling [17, 18, 25, 26]. Only one center offered a short (two-week) intensive inpatient auditory rehabilitation course prior to outpatient training as standard [18, 25]. Program lengths varied between one and three years’ post-implantation [12, 18, 23, 24], with reported session frequency weekly [26], fortnightly [23] or 2-monthly [18, 25], and further appointments given according to need [12, 23]. Two pre-lingually deaf patients received pre-implantation auditory-oral therapy [16]. Audioverbal habilitation programs were diverse, comprising speech production, speech reading, auditory training in quiet and group conversation, comprehension and language development, and telephone use [18, 19, 25, 26], with an emphasis on auditory cues in some centers [16] and combined auditory-visual cues in others [18, 25, 26].

Success was partially attributed to personal motivation [8], with parents acting as facilitators of change in studies of implantation in childhood [12, 26]. Children unable to attend the centers regularly due to geographical distance or comorbidity were disadvantaged [23, 26]. Increased time and labor requirements in the assessment and rehabilitation of patients with Usher syndrome compared to normally sighted patients were reported by three studies [8, 27, 28], with two studies [8, 28] reporting a need for tactile communication techniques and interpreters. One study [29] reported no difficulties in delivering speech therapy despite the visual disturbance, but the extent of visual impairment was less severe in their population.

### Quality Assessment

**Fig. 2:**
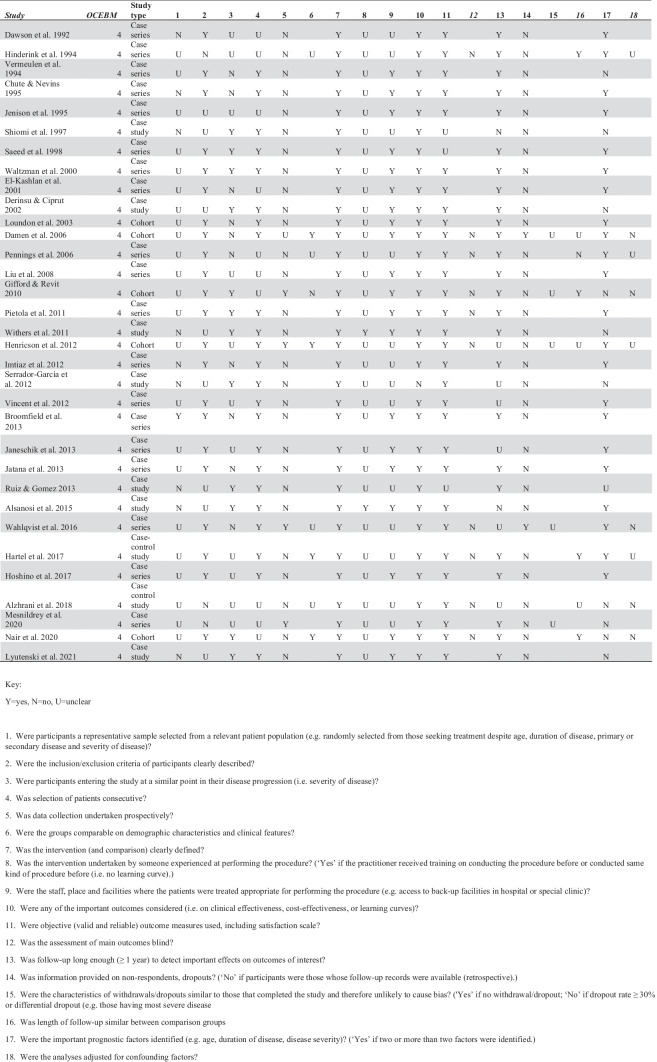
Risk of bias assessment using Brazzelli tool [7] and OCEBM grading [6]. Key: Y = yes, N = no, U = unclear. 1. Were participants a representative sample selected from a relevant patient population (e.g. randomly selected from those seeking treatment despite age, duration of disease, primary or secondary disease and severity of disease)? 2. Were the inclusion/exclusion criteria of participants clearly described? 3. Were participants entering the study at a similar point in their disease progression (i.e. severity of disease)? 4. Was selection of patients consecutive? 5. Was data collection undertaken prospectively? 6. Were the groups comparable on demographic characteristics and clinical features? 7. Was the intervention (and comparison) clearly defined? 8. Was the intervention undertaken by someone experienced at performing the procedure? (‘Yes’ if the practitioner received training on conducting the procedure before or conducted same kind of procedure before (i.e. no learning curve).) 9. Were the staff, place and facilities where the patients were treated appropriate for performing the procedure (e.g. access to back-up facilities in hospital or special clinic)? 10. Were any of the important outcomes considered (i.e. on clinical effectiveness, cost-effectiveness, or learning curves)? 11. Were objective (valid and reliable) outcome measures used, including satisfaction scale? 12. Was the assessment of main outcomes blind? 13. Was follow-up long enough (≥ 1 year) to detect important effects on outcomes of interest? 14. Was information provided on non-respondents, dropouts? (‘No’ if participants were those whose follow-up records were available (retrospective).) 15. Were the characteristics of withdrawals/dropouts similar to those that completed the study and therefore unlikely to cause bias? ('Yes’ if no withdrawal/dropout; ‘No’ if dropout rate ≥ 30% or differential dropout (e.g. those having most severe disease. 16. Was length of follow-up similar between comparison groups. 17. Were the important prognostic factors identified (e.g. age, duration of disease, disease severity)? (‘Yes’ if two or more than two factors were identified.). 18. Were the analyses adjusted for confounding factors?

All included studies were graded at level 4 of the Oxford CEBM Levels of Evidence. Study quality was generally modest with deficiencies largely attributed to retrospective data collection and lack of clarity regarding patient representativeness (Fig. 2). Heterogeneity of reported outcomes precluded meta-analysis.

## Discussion

This review identified post-implantation auditory and QOL outcomes for 186 patients with Usher syndrome, complementing and updating a previous systematic review on cochlear implant outcomes that included literature up to November 2019 [41] and provides an insight into the impact of cochlear implant on patient’s schooling. Our results suggest that good audiometric and quality of life outcomes can be expected in the majority of patients with Usher types 1, 2 and 3. Our review was, however, limited by the quality of available evidence in the literature particularly for patients with Usher types 2 and 3 where only a limited number of studies reported audiometric outcomes.

For patients with Usher type 1, the extent of implant success after implantation in pre-lingually deafened patients was highly variable. Much of this may be accounted for by the effect of patient age at implantation. Several studies of non-syndromic pediatric patients have identified a transition period between the ages of 24–36 months after which implantation results in relatively inferior perceptive and communicative outcomes [3]. Amongst studies of Usher syndrome, Loundon et al. [29] reported better perceptive results in children implanted below the age of nine years. Amongst pre-lingually deaf patients implanted as adolescents or adults, few achieved open-set speech perception [16, 18, 22, 26, 29, 33, 35], consistent with outcomes reported from non-syndromic populations [42]. Auditory outcomes appear similar to those of pre-lingually deaf patients with normal vision [3, 29, 30, 42]. Henricson et al. [40] found that auditory information processing performance in children with Usher type 1 was similar to that of children with normal hearing, except in tests of phonological working memory and lexical skill. In these subtests, they generally performed better than non-Usher cochlear implant users, but poorer than normal hearing and hearing aid-assisted children, likely due to the relatively earlier implantation of children with diagnosed Usher syndrome in their cohort. The improved outcomes with early implantation seen in this review suggests that genetic screening of children who are born profoundly deaf is important to enable children with Usher syndrome to be identified early and allow for effective counselling for patients and their families regarding the treatment options available.

Effects on speech perception and quality of life were more consistent among post-lingually deaf patients with Usher syndrome, with all patients achieving open-set speech recognition. Auditory outcomes are similar to those reported by other studies of post-lingually deafened adults [43]. Among post-lingually deafened adults, QOL benefit of implantation for patients with Usher syndrome was found to be comparable to that of non-Usher patients without visual deficits in two of the studies included in this review [21, 44]. Rehabilitation practices were generally reported poorly, if at all. Janeschik et al. [10] found that in the first three years after implantation, children with Usher syndrome had worse post-implant hearing outcomes compared to other children with hereditary hearing loss but at 48 months after implantation, results between the two groups were comparable. The team attributed this relatively slow improvement in auditory-oral ability to severe progressive visual loss experienced by patients with Usher syndrome. However, the number of Usher syndrome patients in the study were small and rehabilitation efforts were confounded by underlying cognitive deficits and bilingual education in the Usher syndrome group. In a separate study, Hinderink et al. [18] identified a plateau in auditory abilities amongst one adolescent and adults with Usher type 1 beyond 12 months post-implant, consistent with studies of non-syndromic pre-lingually deaf adolescents and adults [45, 46].

None of the studies included in this review identified any correlation between the genotype and post-implantation outcomes. Reported outcome data were sufficiently heterogenous as to preclude meta-analysis of auditory outcomes and quality of life with respect to age at implantation or visual impairment.

Eight studies [12, 17–19, 22, 36–38] reported educational placement. As recent changes in public policy favor the integration of children with disabilities into mainstream schools where patients are more likely to achieve language development comparable to that of normal-hearing peers [47], the extent of language development reported may be an underestimation of contemporary outcomes in children with Usher syndrome and cochlear implants. Whilst several studies report of individual cases that have successfully enrolled at mainstream schools, no studies report both pre- and post- implantation educational placement for the same patient cohort. This highlights a gap in the published literature of whether cochlear implantation not only improves audiometric outcomes but whether this translates into improvements in educational outcomes. The seven studies identified in this review that reported on QOL measures would suggest improvements in educational outcomes to be present as improvements in QOL post-implantation were found in the majority of cases. There was, however, data that suggested that general QOL negatively correlated with age of implantation and speech perception among patients with Usher type 1 [30]. As cochlear implant costs are estimated to be over $1 million US dollars over a lifetime [48], further research into quantifying educational outcomes post-cochlear implantation in this population may be useful for policy makers, clinicians and patients/parents in making an informed decision regarding cochlear implantation.

The modest quality of included studies is a limitation of this review that reflects the low prevalence of Usher syndrome in the general population and varied reporting practices. Though not possible to quantify, we suspect that some reporting bias in favor of extreme results will be present, particularly among case studies and smaller case series. Future studies would benefit from the use of standardized international reporting measures for auditory outcomes and quality of life, and systematic reporting of educational placement, communication preferences, and educational attainment. Studies should also report data regarding non-implanted patients with Usher syndrome assessed at their center to better determine the representativeness of the implanted cohort. Multi-center collaboration may identify larger cohorts of patients with Usher syndrome, from which more confident estimations of effect may be inferred.

## Conclusion

Where reported, cochlear implantation was found to be beneficial in improving sound detection, speech perception, speech intelligibility, and quality of life in the majority of patients with Usher syndrome. As clinical practice has evolved to emphasize early, bilateral implantation and access to oral education it is likely that the outcomes reported in this systematic review underestimate the potential benefits of cochlear implantation among young children with Usher syndrome. Clearly, early identification and implantation is even more important in the prelingually deaf Usher group, as they are likely to proceed to visual loss in time, with resulting multisensory deficits if cochlear implant outcomes are poor due to late implantation.

### Supplementary Information

Below is the link to the electronic supplementary material.

Supplementary file1 (PDF 13 KB)

Supplementary file2 (PDF 71 KB)

Supplementary file3 (PDF 254 KB)

## Data Availability

Not applicable.
