# Supplementary material for: Outcomes of cochlear implantation in Usher syndrome: a systematic review
Source: Eur Arch Otorhinolaryngol. 2023 Nov 6;281(3):1115–29. doi: 10.1007/s00405-023-08304-2 (PMC10858075; doi:10.1007/s00405-023-08304-2)
Supplement: Supplementary file 1 — Supplementary file1 (PDF 13 KB) [file 405_2023_8304_MOESM1_ESM.pdf]

	Subtype		
	I	II	III
SNHL	Congenital<br>Severe-to-profound	Congenital<br>Mild-to-severe<br>May be progressive.	Normal at birth<br>Variable timing of onset late childhood to adulthood, progressive.
RP	Symptom onset by 10 years of age	Symptom onset in 2nd decade	Symptom onset in 2nd decade
VD	Present, congenital	Absent	May be present or absent
Abbreviations: sensorineural hearing loss (SNHL), retinitis pigmentosa (RP), vestibular dysfunction (VD).			

Online Resource 1 - Characteristics of Usher syndrome subtypes.

Article Title: Outcomes of cochlear implantation in Usher syndrome: a systematic review

Journal name: European Archives of Oto-Rhino-Laryngology and Head & Neck

Authors: HL Cornwall[1], CM Lam[1], D Chaudhry[2], J Muzaffar[3,4], P Monksfield[4], ML Bance[3,5]

Affiliations: [1] Cardiff and Vale University Health Board, Cardiff, UK. [2] College of Medical and Dental Sciences, University of Birmingham, Birmingham, UK. [3] Department of Clinical Neurosciences, University of Cambridge, Cambridge, UK. [4] Department of Otolaryngology, University Hospitals Birmingham NHS Foundation Trust, Birmingham, UK. [5] Department of Otolaryngology, Addenbrooke's Hospital, Cambridge University Hospitals NHS Foundation Trust, Cambridge, UK

Corresponding author: Professor Manohar L Bance, email: mlb59@cam.ac.uk
