# Supplementary material for: Outcomes of cochlear implantation in Usher syndrome: a systematic review
Source: Eur Arch Otorhinolaryngol. 2023 Nov 6;281(3):1115–29. doi: 10.1007/s00405-023-08304-2 (PMC10858075; doi:10.1007/s00405-023-08304-2)
Supplement: Supplementary file 2 — Supplementary file2 (PDF 71 KB) [file 405_2023_8304_MOESM2_ESM.pdf]

Database	Search terms
Pubmed MEDLINE	(cochlear implants [Mesh] OR cochlear implantation [Mesh] OR implant* OR hearing aid [Mesh] OR electro-acoustic OR electroacoustic OR electric acoustic OR electric-acoustic) AND (Usher syndrome [Mesh] OR Usher* [ti] OR Usher)
Ovid EMBASE	(exp cochlear implantation/ OR cochlear implant*.mp. OR exp hearing aid/ OR hearing aid.mp.) AND (usher syndrome.mp. OR exp Usher syndrome/ OR usher.mp.)
Web of Science	ALL=(cochlear implants OR cochlear implantation OR implant* OR hearing aid OR electro-acoustic OR electroacoustic OR electric acoustic OR electric-acoustic) AND ALL=(Usher syndrome OR Usher* OR Usher)
Cochrane<br>CENTRAL	#1 MeSH descriptor: [Cochlear Implants] explode all trees<br>#2 cochlear implant*<br>#3 MeSH descriptor: [Cochlear Implantation] explode all trees<br>#4 implant*<br>#5 electro-acoustic OR electroacoustic OR electric acoustic OR electric-acoustic<br>#6 MeSH descriptor: [Hearing Aids] 1 tree(s) exploded [tree number 2 – sensory aids]<br>#7 hearing aid*<br>#8 MeSH descriptor: [Usher Syndromes] explode all trees<br>#9 usher<br>#10 (#1 OR #2 OR #3 OR #4 OR #5 OR #6 OR #7) AND (#8 OR #9)
ClinicalTrials.gov	'Usher Syndromes'

Online Resource 2 - Complete search strategy

Article Title: Outcomes of cochlear implantation in Usher syndrome: a systematic review

Journal name: European Archives of Oto-Rhino-Laryngology and Head & Neck

Authors: HL Cornwall[1], CM Lam[1], D Chaudhry[2], J Muzaffar[3,4], P Monksfield[4], ML Bance[3,5]

Affiliations: [1] Cardiff and Vale University Health Board, Cardiff, UK. [2] College of Medical and Dental Sciences, University of Birmingham, Birmingham, UK. [3] Department of Clinical Neurosciences, University of Cambridge, Cambridge, UK. [4] Department of Otolaryngology, University Hospitals Birmingham NHS Foundation Trust, Birmingham, UK. [5] Department of Otolaryngology, Addenbrooke's Hospital, Cambridge University Hospitals NHS Foundation Trust, Cambridge, UK

Corresponding author: Professor Manohar L Bance, email: mlb59@cam.ac.uk
