# Supplementary material for: Outcomes of cochlear implantation in Usher syndrome: a systematic review
Source: Eur Arch Otorhinolaryngol. 2023 Nov 6;281(3):1115–29. doi: 10.1007/s00405-023-08304-2 (PMC10858075; doi:10.1007/s00405-023-08304-2)
Supplement: Supplementary file 3 — Supplementary file3 (PDF 254 KB) [file 405_2023_8304_MOESM3_ESM.pdf]

Reference	Patients (implants)	Usher type	Mean age at 1st implant in years (range)	Pre-operative data	Post-operative data	Mean follow-up in months (range)
Dawson 1992[26]	3 (3)	N.S.†	18.2 (14.9-20.1)	**Communication mode**<br>Cueing supplement (2 patients), total communication (1 patient).<br><br>**Audiometry**<br>Unaided pure tone hearing thresholds (dB HL) at 0.125, 0.25, 0.5, 0.75, 1, 1.5, 2, 3, 4, 6, 8 kHz (R/L):<br>Patient 6: N.T./>75, >90/>90, >110/>110 at frequencies 0.5-6 kHz, >100/>100.<br>Patient 7: N.T./>75, >90/>90, >110/>110 at frequencies 0.5-6 kHz, >100/>100	**Speech perception**<br>Results given for most recent follow-up testing period. Scores presented for patients 6, 7, 10, in order.<br><br>CLOSED-SET<br>MSTP test scores (n/12): 3, 9*, 6*<br>Picture Vocabulary Test scores (n/12): 1, 8*, 4*<br>NUCHIPS Test scores (n/50): 19, 22, 15<br>Vowel Length Subtest scores (n/50): 45*, 48, 37<br>Vowel Place Subtest scores (n/50): 42*, 41, 33<br>Consonant Voicing Subtest scores (n/50): 45*, 48, 45*<br>Consonant Manner Subtest scores (n/50): 33*, 40, 40<br>Consonant Place Subtest scores (n/50): 32*, 29, 26<br>11-Vowel scores (n/44): 14 (32%), 13 (30%), 11 (25%) [all scores exceed chance]	29 (17-38)

				Patient 10: N.T./N.T., 90/95, 110/>120, 120/>120, >120/>120 at frequencies 1-4 kHz, >110/>110, >100/>100.<br><br>**Speech perception**<br>With hearing aids. Scores presented for patients 6, 7, 10, in order. N.T. = not tested.<br><br>CLOSED-SET<br>MSTP test scores (n/12): 2, 0, 0.<br>Picture Vocabulary Test scores (n/12): 1, 0, 0<br>NUCHIPS Test scores (n/50): 14, N.T., 17<br>Vowel Length Subtest scores (n/50): 37, N.T., 41	[* = statistically significant improvement in score, P<0.05]<br><br>OPEN-SET<br>AB Word Test Phoneme scores [lipreading/lipreading+hearing/hearing] (n/30): 14/19/2, 16/18*/5, 9/16/N.T.<br>BKB Sentence Test scores [lipreading/lipreading+hearing/hearing] (n/50): 16/20*/N.T., 20/37*/0, 4/6/N.T.<br>[*average postoperative lipreading+hearing score is statistically greater (P<0.05) than average postoperative lipreading score]<br><br>**Other**<br>Schooling:<br>Child 7 attends an auditory/oral school where cueing supplement to lipreading is used alongside auditory/oral.<br>Patient 10 now works as an apprentice cabinet maker not relying entirely on total communication in work.	

				Vowel Place Subtest scores<br>(n/50): 31, N.T., 38<br>Consonant Voicing Subtest scores<br>(n/50): 26, N.T., 24<br>Consonant Manner Subtest scores<br>(n/50): 23, N.T., 32<br>Consonant Place Subtest scores<br>(n/50): 19, N.T., 30<br><br>OPEN-SET<br>AB Word Test Phoneme scores<br>[lipreading/lipreading+hearing]<br>(n/30): N.T./N.T., N.T./N.T., 12/9<br>BKB Sentence Test scores<br>[lipreading/lipreading+hearing]<br>(n/50): 20/23, N.T./N.T.,<br>N.T./N.T.		

				No results for patient 7 as no aided speech reception thresholds below the maximum output of their hearing aid.<br><br>**Other**<br>Patient 10 attended a school for deaf children using total communication, then moved to a deafness unit in a mainstream school.		
Hinderink 1994[18][§] / Vermeulen 1994[25][§]	4 (5)	USH1	21.2 (13.4-28.9)	**Communication mode**<br>All patients capable of lip-reading. Case 2 could use Total communication.<br><br>**Audiometry**	**Audiometry**<br>1-year Free field hearing thresholds (dB SPL) at 0.25, 0.5, 1, 2, 4, 8 kHz:<br>Case 1 - single channel implant: 40, 65, 60, 60, 75, 85<br>Case 1 - 22-channel implant: 40, 40, 35, 35, 35, 35<br>Case 2 - 22-channel implant: 50, 65, 50, 55, 50, 60<br>Case 3 - single channel implant: 40, 45, 50, 60, 45, 35<br>Case 4 - 22-channel implant: 45, 40, 40, 30, 30, 30	24 (24)

				Unaided best ear pure tone audiometry hearing thresholds (dB SPL) at 0.25, 0.5, 1, 2, 4, 8 kHz:<br>Case 1: >110, >120, >120, >120, >110, >100<br>Case 2: 80, 100, 110, >120, >110, >100<br>Case 3: 95, 120, >120, >120, >110, >100<br>Case 4: >110, >120, >120, >120, >110, >100<br><br>Free field hearing thresholds with hearing aids (dB SPL) at 0.25, 0.5, 1, 2, 4, 8 kHz:<br>Case 1: 65, 65, >120, >120, >100, >100<br>Case 2: 50, 65, 65, 80, >100, >100<br>Case 3: Not measured.	**Speech perception**<br>MTS test, dutch:<br>Case 1 - above chance in all domains<br>Case 2 - above chance in syllable detection and word recognition<br>Case 3 - above chance in syllable and word recognition<br>Case 4 - above chance in syllable and word recognition<br><br>AN test battery<br>Case 1 - above chance in all tests<br>Case 2 - above chance in short vowel identification, number of syllables, male/female/child, and environmental sounds<br>Case 3 - above chance in all areas except sentence accent identification<br>Case 4 - above chance on vowel recognition and pattern recognition except sentence accent identification.<br><br>CDT task (lipreading skill), words per minute at 12 months (visual/auditory-plus-visual):	

				Case 4: 75, 110, >110, >110, >100, >100<br><br>**Other**<br>All patients educated at a School for the Deaf.	Case 1 - 1-channel: 40/60; 22-channel: 50/56 (max score 68)<br>Case 2 - 20/22 (max score 56)<br>Case 3 - 25/27 (max score 40 due to visual impairment)<br>Case 4 - 22/22 (max score 60)<br>Only a small improvement in speechreading when using the CI.<br>No open-set speech recognition in auditory-only condition in any patient.<br><br>**Other**<br>Case 1 - Daily CI user (3-12h/day)<br>Case 2 - Daily CI user, enrolled at a mainstream school, interpreter assistance.<br>Case 4 - Daily CI user >12h/day.<br>All patients recognised environmental sounds after 3 months.<br>Communication, orientation and mobility were less stressful.	
Chute & Nevins 1995[36]	3 (3)	N.S.	8.5 (6.5-9.5)	**Speech perception**<br>(with vibrotactile device, CH/BS)<br>Closed-set tests (NU-CHIPS, GASP, TAC): could not test	**Speech perception**<br>(at last follow-up, CH/BS)<br>NU-CHIPS at 2 years: 50%, 28%<br>GASP (words): 50%, 0%	22 (6-36)

				ESP category: 1 (CH)<br>Minimal pairs test: N.T. (CH)<br>WIPI: 48% (visual-only), 64% (auditory-visual) (BS)<br>MTS: did not test<br><br>**Speech intelligibility**<br>CID SPINE category: unintelligible	GASP (sentences): 90%, 0%<br>TAC: 7, 2<br>ESP category: 5 (CH)<br>Minimal pairs test: 84% (CH)<br>WIPI: 80% (visual-only), 76% (auditory-visual) (BS)<br>MTS: 25% (words), 68% (stress) (BS)<br><br>**Speech intelligibility**<br>CID SPINE category: fair intelligibility, unintelligible<br><br>**Other**<br>Schooling: CH is an A-grade student in a mainstream class for gifted children with sign-language support from an interpreter and a teacher of visually-impaired students. BS is an average-performing child in a school for deaf children that uses Total communication and is committed to auditory training.<br>All 3 detect environmental sounds and respond to their name when called.<br>BS and BR are full-time CI users.	

Jenison 1995[17]	2 (2)	USH1	Children (likely between ages 8-12)	**Audiometry**<br>Unaided hearing thresholds (with traditional hearing aids) in dB HL for L/R ears at 0.25, 0.5, 1, 2, 4 kHz:<br>Case 1: 90/90 (37.5), 110/110, >130/>130, >130/>130, >130/>130<br>Case 2: 95/100 (45), >130/120 (70), >130/>130 (92), >130/>130, >130/>130.<br><br>**Speech perception**<br>ESP Pattern set of 12: 40% (case 1), 40% (case 2).<br>NU-CHIPS set of 4: 25% (case 1), 15% (case 2).<br><br>**Other**	**Audiometry**<br>Sound field hearing thresholds (dB HL) at 0.25, 0.5, 1, 2, 4 kHz:<br>Case 1: 25, 40, 35, 35, 40<br>Case 2: 20, 25, 25, 35, 30.<br><br>**Speech perception**<br>ESP at 36 months: Case 1 - 75-80% (pattern set of 4-12, mono set of 4), 50% (spondee). Case 2 - 100% (pattern, set of 4), 25-60% (pattern set of 12, spondee and mono set of 4).<br>NU-CHIPS set of 4 at 36 months: 25% (case 1), 28% (case 2).<br>GASP open set: 15% (case 1, 24 months), 10% (case 2, 6 months).<br>Case 1 can identify words based on stress pattern but has not yet reached a passing score on spectral tasks.<br>Case 2 can identify words based on stress pattern when the set size is controlled but has no consistent word identification based on spectral content.<br><br>**Other**	36 (36)

				Both attended mainstream school with an interpreter.	Both are full-time CI users.	
Shiomi 1997[31]	1 (1)	N.S.‡	Adult	**Communication mode**<br>Palm writing.<br>No residual hearing.	**Speech perception**<br>Closed-set testing:<br>vowels = 100%<br>consonants = 52%<br><br>**Other**<br>Understands conversation in daily life and successfully uses the implant every day.	3
Saeed 1998[28]	1 (1)	USH1	<5	Prelingual severe or profound deafness.	**Speech perception**<br>Can identify LING sounds across the speech frequency range (0.3-4 kHz).<br><br>**Other**<br>Can follow two- or three- item instructions.<br>Improvement in general self-confidence, perception of environmental sounds and lip-reading skill.	>36

Waltzman<br><br>2000[37]	1 (1)	N.S.	4.4	Profoundly hearing impaired.	**Communication mode**<br><br>Oral<br><br><br>**Speech perception**<br><br>CLOSED-SET tests<br><br>CID (central institute for the deaf) ESP (early speech perception)<br><br>test: 4<br><br>NU-CHIPS: 100%<br><br><br>OPEN-SET tests<br><br>GASP word test: 100%<br><br>PBK test: 70% (words), 83% (phonemes)<br><br>MLNT: could not perform<br><br>LNT: could not perform<br><br>Common phrases sentence test: could not perform<br><br><br>**Other**<br><br>Mainstream school.	60

El-Kashlan 2001[27]	1 (1)	USH1	3.5	**Speech perception**<br>(with hearing aids)<br>ESP score: 0%	**Speech perception**<br>ESP score: 100% at 1 year<br>GASP: 75% at 1 year<br>Open-set recognition, common phrases: 100%<br>Open-set CID sentences: 88% (auditory-only)<br><br>**Other**<br>Expressive vocabulary = 100 words at 1 year.	108
El-Kashlan 2001[27]	1 (1)	N.S.†	32	**Communication mode**<br>Sign language, no oral vocabulary.<br><br>**Speech perception**<br>CID sentences: 0%	**Speech perception**<br>Improved pattern perception, beginning to develop closed-set skills for familiar names.<br><br>**Other**<br>Greater awareness of environmental sounds. Increased anxiety when the implant was not turned on.	12
Derinsu & Ciprut 2002[8]	1 (1)	N.S.‡	52	**Communication mode**<br>Palm writing, Braille alphabet<br><br>**Audiometry**	**Communication mode**<br>Auditory-verbal, able to converse by telephone.<br><br>**Audiometry**	48

				Pure-tone audiometry dB SPL R/L:<br>250 Hz - 110/93<br>500 Hz - 120/113.<br>Speech detection thresholds dB SPL R/L: 115/105.<br><br>**Speech perception**<br>No measurable word recognition threshold.	Sound field warble tone thresholds 30-40 dB SPL across range 250 Hz - 4 kHz.<br><br>**Speech perception**<br>Open set sentence recognition 72% at 6 months, 100% at 4 years.<br>Open set monosyllabic word recognition 56% at 6 months, 80% at 4 years.<br><br>**Other**<br>Patient reported feeling less dependent on relatives, more confident, social and happy.<br>Full-time CI user.	
Loundon 2003[29]	11 (11)	USH1	6.8 (1.6-20)	**Speech perception**<br>Closed set (mean): 0%<br>Open set (mean): 0%<br><br>**Speech intelligibility**<br>Speech production: complex sentences (2 patients), spared	**Speech perception**<br>Closed set (mean): 81.8% (range 0%-100%)<br>Closed set (individual data points, %): 100, 100, 100, 0, 100, 100, 100, 50, 100, 100, 50<br><br>Open set (mean): 33.2% (range 0%-90%)	46.9 (9-96)

				words (1 patient), no production (8 patients).	Open set (individual data points, %): 50, 25, 75, 0, 25, 50, 90, 0, 0, 50, 0<br><br>**Speech intelligibility**<br>Speech production: complex sentences (4 patients), simple sentences (2 patients), spared words (4 patients), no production (1 patient).	
Loundon 2003[29]	1 (1)	USH3	44	**Speech perception**<br>Closed set: 0%<br>Open set: 0%<br><br>**Speech intelligibility**<br>Speech production: complex sentences	**Speech perception**<br>Closed set: 100%<br>Open set: 75%<br><br>**Speech intelligibility**<br>Speech production: complex sentences	108
Loundon 2003[29]	1 (1)	N.S.†	3	**Speech perception**<br>Closed set: 0%<br>Open set: 0%<br><br>**Speech intelligibility**	**Speech perception**<br>Closed set: 100%<br>Open set: 90%<br><br>**Speech intelligibility**	60

				Speech production: no production	Speech production: complex sentences	
Pennings 2006[30][§] / Damen 2006[33][§]	14 (15)	USH1	12.4 (3.5-30.4)	**Speech perception**<br>Mean T0 EHL when implanted aged:<br><10y = 122 dB HL,<br>10-19y = 128 dB HL<br>>19y = 125 dB HL<br><br>**Other**<br>All patients were profoundly deaf.	**Speech perception**<br>Mean last-visit EHL (by Pennings et al.) when implanted aged:<br><10y = 84 dB HL (5/7 patients significantly improved EHL),<br>10-19y = 97 dB HL (1/3 patients significantly improved EHL),<br>>19y = 115 dB HL (no patients significantly improved EHL)<br>EHL scores similar to those of congenitally-deaf patients with normal vision.<br>Mean EHL at last follow-up (by Damen et al.): 95.8 dB HL (range 70-120 dB HL)	60 (24-120) / 109 (36-188)
Liu 2008[16]	9 (9)	USH1	5.4 (2-11)	**Communication mode**<br>Oral (2 patients), sign (5 patients), total (2 patients).<br><br>**Audiometry**<br>At frequencies 0.5, 1, 2, 4 kHz, hearing threshold (dB HL) was >115 (1 patient), >110 (7 patients), 100 (1 patient).	**Communication mode**<br>Auditory-oral (2 patients), lip reading or total communication (7 patients). All are implant users.<br>QACIU score: 3 (7 patients), 1 (2 patients).<br><br>**Audiometry**<br>At frequencies 0.5, 1, 2, 4 kHz hearing threshold (dB HL, mean, range) was: 45.6 (35-60), 42.8 (30-65), 41.7 (30-60), 38.9 (25-60).	21.3 (12-24)

				**Speech perception**<br>CAP score: 0 for all patients.	**Speech perception**<br>Closed set monosyllable recognition (mean, 8 patients):<br>63.5%, range 20%-100% (all patients).<br>80%, range 70%-100% (3 patients implanted aged <4y).<br>54%, range 20-100% (5 patients implanted aged >6y).<br><br>Open-set monosyllable recognition (7 patients):<br>With lip reading: 0% (4 patients, all implanted aged >6y), 80-85% (3 patients, 2 <4y, 1 >6y).<br>No lip reading: 0% (6 patients), 60% (1 patient, implanted <4y).<br><br>CAP score (mean): 3.8 (range 2-4) at 12-24 months.	
Gifford & Revit 2010[34]	1 (1)	N.S.	15.9	N.S.	**Speech perception**<br>CNC Word-Recognition score in quiet (% correct): 76<br>HINT Sentence Recognition Score in quiet (% correct): 94<br>R-SPACE speech reception threshold (dB SNR): 17 (behind the ear mic), 12.5 (T mic)	25

Pietola 2012[21]	19 (19)	USH3	41 (10-64)	**Audiometry**<br>Mean PTA (0.5-4k Hz) in (dB HL):<br>104 +/- SD 10 (5-10 years before implantation)<br>110 +/- SD 8 (12 months before implantation, no aids)<br>58 +/- SD 11 (12 months before implantation, with hearing aids)<br><br>**Speech perception**<br>Recognition of recorded Finnish bisyllabic words, aided with hearing aids:<br>17% +/- SD 25% (5-10 years before implantation)<br>4% +/- SD 9% (0-12 months before implantation)	**Audiometry**<br>Mean PTA (0.5-4k Hz) in (dB HL):<br>34 +/- SD 9 (12 months post-implant)<br>[p<0.01 compared to pre-implant HA-aided PTA, Student's t-test]<br><br>**Speech perception**<br>Recognition of recorded Finnish bisyllabic words 6-18 months after implantation: 52% +/- SD 33%.<br>[p<0.001 compared to 0-12 months pre-implant value]<br><br>**Other**<br>15 patients used their CI 12-16h/day or were full-time users of their CI. 2 patients used their CI <12h/day. 2 patients did not provide data.<br><br>11/18 patients had seldom or no tinnitus.<br>8/13 patients reported that the CI reduced or extinguished their tinnitus.	12

Withers 2011[9]	1 (2)	N.S.	63	**Audiometry**<br>Pure tone audiometry dB SPL thresholds for ears L/R:<br>250 Hz - 80/75<br>500 Hz - 75/75<br>750 Hz - 95/90<br>1 kHz - 110/120<br>1.6 to 6 kHz - 120/120<br>8 kHz - 100/100<br><br>**Speech perception**<br>Right ear with hearing aid, sound at 65 dB SPL.<br>CUNY sentences = 0%<br>CNC words = 0%	**Speech perception**<br>12 month follow-up from second implantation (56 months from 1st): Left only / Right only / Bilateral<br>(sound at 65 dB SPL)<br>CUNY sentences = 95% / 82% / 100%<br>CUNY in noise (4 talker babble) + 10dB SNR 65/55 = - / - / 70%<br>CNC words = 48% / 36% / 36%<br>Vowels = 80% / 52% / 56%<br>Consonants = 68% / 54% / 56%<br>Phonemes = 72% / 53% / 56%<br><br>**Speech production**<br>'Friends had commented that she was speaking more naturally [following the second implant].'	56
Henricson 2012[40]	7 (13)	USH1	2.1 (0.8-4)	N.S.	**Communication mode**<br>Spoken Swedish (5 patients), sign-supported Swedish (1 patient), Swedish sign language and spoken Swedish (1 patient).	100.6 (70-143)

					**Other**<br>Sound Information Processing Systems test battery.<br>Phonological representations: mean 15.8 (SD: 11.9-19.7)<br>Non-word discrimination: mean 7.2 (SD: 6.1-8.2)<br>Phoneme identification: mean 8.8 (SD: 5.1-12.5)<br>Outcomes compared to children with normal hearing (NH), hearing impairment with hearing aids (HA), and non-Usher children with cochlear implants (CI).<br><br>General working memory (WM) similar to HA and NH, better than CI. Visual WM scores over a wide range of performance. Phonological WM generally worse than HA and NH, similar to or better than CI. 2 of 7 scored similar to or better than NH and HA.<br><br>Performance on phonological and lexical skill tasks tended to be in line with expected performance for NH and HA groups for children implanted before age 2 years, but closer to that of non-syndromic CI users when implanted at later ages.	

Imtiaz 2012[19]	3 (3)	USH1	3.1 (1.3-6)	**Audiometry**<br>Unaided hearing thresholds (dB HL) at 0.25, 0.5, 1, 2, 4 kHz:<br>Patient 1: 100, 105, >110, >110, >110 (symmetrical L and R sides, age 5y)<br>Patient 2: 100, 105, 110, >120, >120 (sound field testing, age 1y)<br>Patient 3: 80, 90, 100, 102, 108 (sound field testing, age 1y)<br><br>Free field hearing thresholds with hearing aids (dB HL) at 0.25, 0.5, 1, 2, 4 kHz:<br>Patient 1: 53, 60, 75, 75, 95, >103 (age 5y)<br>Patient 2: 80, 90, 100, >115, >115 (age 1.5y)	**Audiometry**<br>Hearing thresolds (dB HL) at 0.25, 0.5, 0.75, 1, 1.5, 2, 3, 4, 6 kHz:<br>Patient 1: 15, 10, 10, 10, 5, 10, 10, 15, 15 (age 18y)<br>Patient 2: 25, 15, 25, 15, 10, 20, 15, 20, 20 (age 9y)<br>Patient 3: 15, 15, not done, 10, 5, 15, 10, 15, 20 (age 6y)<br><br>**Speech perception**<br>All 3 patients can comprehend open-set speech, answer Wh-questions from an open set, participate effectively in group conversation, and discriminate on the telephone with familiar (patient 1) or unfamiliar (patients 2 and 3) persons.<br><br>**Other**<br>Schooling: patient 1 is undertaking a bachelor's degree in business administration (age 18), patients 2 and 3 are enrolled in mainstream schools (age 9 and 6).	95 (56-144)

				Patient 3: 75, 85, 90, >100, >100 (age 1.5y)		
Serrador-García 2012[14]	1 (1)	N.S.	Adult	N.S.	**Audiometry**<br>Pure-tone audiometry dB HL right side, implant on(off):<br>125 Hz - N.S.(>110), 250 Hz - 50(>110), 500 Hz - 40(>110), 1k Hz - 60(>110), 2k Hz - >110(>110), 4kHz - >110(>110), 8k Hz - >110(>110)	N.S.
Vincent 2012[38]	2 (4)	N.S.	3.6 (2.5-4.7)	N.S.	**Speech perception**<br>(Using Boorsma word lists; patient 1, patient 2)<br>Speech in quiet (% correct): right - 60, 80; left - 100, 60; bilateral - 100, 65<br>Speech in noise (% correct): right - 80, 70; left - 60, 80; bilateral - 100, 50.<br><br>**Other**<br>Both children attend mainstream schools.	64.8 (27.6-102)
Broomfield 2013[23]	9 (N.S.)	N.S.	6.1 (1.3-18)	**Other**<br>All patients had severe to profound hearing loss which was	**Communication mode**<br>5 patients used speech only, 2 patients used combination speech and sign, 2 patients used sign only.	123 (58-203)

				congenital in 8 cases and progressive in one case.<br>One patient had mild cognitive delay.	**Speech perception**<br>Geers & Moog speech perception score was 6 (open set word recognition) in 4 patients, 5 (identification of words that differ for one consonant) in one patient, and 4 (identification of words that differ by one vowel) for 3 patients. One patient was not tested (non-user).<br>BKB % (range 0-100) was tested in 4 patients with outcomes: 68, 92, 94, 94.<br><br>**Other**<br>7 patients were full-time users of their CI, 1 part-time, and 1 non-user.	
Janeschik 2013[10]	7 (13)	N.S.	4.3 (N.S.)	**Audiometry**<br>All patients had a wave V click threshold of 90 dB nHL or more in BERA or a speech perception score <=30% in free field	**Speech perception**<br>Mainzer speech perception test (mean score) at x months post-op from first implant:<br>6 months = 20%<br>12 months = 42%<br>18 months = 55%	60

				audiometry with hearing aids (presented at 65 dB SPL).<br><br>**Speech perception**<br>Mainzer speech perception test (mean score) = 7%	24 months = 67%<br>30 months = 70%<br>36 months = 77%<br>48 months = 88%<br>60 months = 95%	
Jatana 2013[35]	26 (38)	N.S.	3.3 (0.5-11.6)	N.S.	**Communication mode**<br>Oral (15 patients), Total/primarily oral (3 patients), Total/primarily manual (6 patients), Augmentative (2 patients).<br><br>**Speech perception**<br>Sound detection (2 patients, complicated by unrelated developmental delay), open-set discrimination (24 patients).<br>Highest level word or sentence perception (in ascending order of difficulty):<br>MLNT easy (2 patients): 33%, 8%<br>MLNT hard (2 patients): 48%, 83%<br>LNT easy (2 patients): 67%, 36%<br>LNT hard (2 patients): 36%, 92%	93.6 (10-187.2)

					PBK word (4 patients): 52%, 80%, 80%, 72%<br>HINT in quiet (12 patients): 88%, 100%, 90%, 100%, 93%, 100%, 96%, 96%, 96%, 96%, 64%, 92%	
Ruiz & Gomez 2013[15]	1 (2)	USH2	33.3	**Other**<br>Deaf in the left ear before implantation.	**Audiometry**<br>PTA on left side (16 months post-implant) = 26 dB HL<br>PTA on right side (25 months post-implant) = 35 dB HL<br><br>**Speech perception**<br>Open set recognition:<br>Everyday sentences score at 65 dB SPL = 20% (left) and 100% (right).	25
Alsanosi 2015[13]	1 (2)	N.S.	0.4	**Other**<br>Profound sensorineural deafness detected during neonatal hearing screening. No benefit from 3-month hearing aid trial.	**Audiometry**<br>Common sound field aided hearing thresholds at 7 months post-implant (age 12 months):<br>15 dB HL at 250 Hz<br>25 dB HL at 500-8000 Hz<br><br>**Speech perception**<br>CAP score: 5 at 8 months post-processor fitting (age 14 months).	8

					**Speech intelligibility**<br>Parents report 10 functional words and a wide variety of sounds.<br><br>**Other**<br>LittlEars Auditory Questionnaire score 30/35 aged 14 months.	
Wahlqvist 2016[11]	3 (3)	USH3	N.S.	N.S.	**Audiometry**<br>PTA4 (in dB HL) on the best ear (with CI turned <u>off</u>):<br>101, 96, 105.	N/A
Hartel 2017[20]	8 (8)	USH2a	59 (47-73)	**Audiometry**<br>Best ear only, unaided.<br>Mean PTA (0.25-2k Hz): 84 dB HL (SD: 16 dB HL)<br>Mean PTA (0.5-4k Hz): 98 dB HL (SD: 17 dB HL)<br><br>**Speech perception**	**Audiometry**<br>Mean PTA (0.5-4k Hz) of residual hearing, unaided: 116 dB HL (SD: 10 dB HL) (4 patients).<br>Mean PTA with implant: 34 dB HL (SD: 18 dB HL).<br>Neither were statistically different to scores of a non-USH2A control group.<br><br>**Speech perception**<br>65 dB SPL NVA open speech recognition test. 7 patients. Data not available for 1 patient.	12 (12)

				With bilateral hearing aids. 65 dB SPL NVA open speech recognition test.<br>Phoneme score, mean: 41% (range 7-74%)	Phoneme score, CI-only, mean: 70% (range 62-95%)<br>Phoneme score, best-aided, mean: 87% (range 70-95%).<br><br>[Best-aided: With CI and contralateral hearing aid (8 patients). Also with additional hearing aids of electroacoustic stimulation in the ear that received a cochlear implant (2 patients).]	
Hoshino 2017[22]	10 (10)	USH1	18.9 (5-49)	**Communication mode**<br>Oral (3 patients), Oral/Sign (1 patient), Sign/Oral (1 patient), Sign only (4 patients), No language (1 patient).<br><br>**Audiometry**<br>Mean PTA with hearing aids (range) = 115 dB SPL (83.75-130 dB SPL).<br><br>**Speech perception**	**Communication mode**<br>Oral (2 patients), Oral/Sign (2 patients), Sign/Oral (1 patient), Sign only (5 patients).<br><br>**Audiometry**<br>Mean PTA with cochlear implant (range) = 38.1 dB SPL (21.25-50 dB SPL).<br><br>**Speech perception**<br>Vowels = 53%, 20%, 15%, 60% (4 patients).<br>4 choice = 58% (1 patient).<br>Closed set = 40%, 30%, 10% (3 patients).<br>Not tested (2 patients).	12 (12)

				Vowels = 13.3%, 26.7%, 46% (3 patients).<br>4 choice = 56% (1 patient).<br>Not tested (6 patients).<br><br>**Other**<br>Mainstream school: 4 patients.<br>Special school: 6 patients.	**Other**<br>Non-user at 12 months post-implant: 2 patients. A third patient abandoned use of their CI subsequently.	
Alzhrani 2018[12]	9 (N.S.)	N.S.	Children (likely <5 years)	N.S.	**Communication mode**<br>Speech (all 9 patients)<br><br>**Audiometry**<br>PTA 0.5-8k Hz (mean): 23.7 (range 15-35)<br>Individual patient PTAs: 30, 23, 25, 35, 30, 20, 15, 20, 15<br><br>**Speech perception**<br>CAP (mean): 7.6 (range 5-9)<br>Individual CAP scores: 9, 6, 7, 5 (reimplanted), 9, 9, 7, 7, 9<br>Assessed by parents or speech pathologist.	N.S.

					**Speech intelligibility**<br>SIR score (mean): 4.9 (range 4-5)<br>Individual SIR scores: 5, 4, 5, 5, 5, 5, 5, 5, 5.<br>Assessed by parents or speech pathologist.<br><br>**Other**<br>Schooling: mainstream (3 patients), hearing-impaired unit in a mainstream school (3 patients), pre-formal education age (2 patients), no data (1 patient).	
Mesnildrey 2020[32]	1 (1)	N.S.†	7	**Other**<br>Pre-lingually deaf.	**Speech perception**<br>PBK open set = 60%	156
Nair 2020[24]	27 (N.S.)	N.S.	2.9 (0.9-4.7)	**Other**<br>All patients had severe to profound sensorineural hearing loss.	**Speech perception**<br>CAP score (mean): 1.4 at 3 months, 2.03 at 6 months, 3.33 at 9 months, 4.4 at 12 months.<br><br>**Speech intelligibility**<br>SIR score (mean): 1.06 at 3 months, 2.03 at 6 months, 2.4 at 9 months, 4.3 at 12 months.	12 (12)

					CAP and SIR were statistically significantly better in the control group than in the Usher group (P<0.05).<br>CAP and SIR scores significantly improved with time (P<0.05).	
Lyutenski 2021[39]	1 (2)	N.S‡	38	**N.S.**	**Speech perception**<br>Hochmair-Schulz-Moser sentences test in noise: 45% at 6 months, 73.58% at 1 year, 31% at 3 years, 95% at 3 months after reimplantation.<br>German Freiburg monosyllabic word test in quiet: 70% at 6 months, 60% at 1 year, 45% at 3 years, 90% at 3 months after reimplantation.<br>**Other**<br>Med El Hearing Implant Sound Quality Index Questionnaire ($HISQUI_{19}$) score: 95 after reimplantation.	39

Notes: pre-lingual deafness (†), post-lingual deafness (‡), studies published from same centre, likely overlapping dataset (§).

Abbreviations: decibels hearing loss (dB HL), decibels sound pressure level (dB SPL), pure tone average (PTA), calculation of the pure tone average for the frequencies 0.5, 1, 2, and 4 kHz (PTA4), categories of auditory performance (CAP), speech intelligibility rating (SIR), qualitative assessment of cochlear implant use (QACIU), equivalent hearing level (EHL), monosyllable-trochee-spondee test (MTS), Antwerp-Nijmegen (AN), Connected Discourse Tracking (CDT), Bamford-Kowal-Bench sentence test (BKB), standardized Dutch open speech recognition test consisting of monosyllabic wordlists (NVA), monosyllable-spondee-trochee-polysyllable test (MSTP), Northwestern

University Children's Perception of Speech (NUCHIPS), Arthur Boothroyd (AB), Geers & Moog Early Speech Perception test (ESP), Glendonald Auditory Screening Procedure (GASP), City University of New York (CUNY), Consonant Nucleus Consonant (CNC), Hearing in Noise Test (HINT), Meaningful Lexical Neighbourhood Test (MLNT), Lexical Neighbourhood Test word test (LNT), Phonetically Balanced Kindergarten word test (PBK), Test of Auditory Comprehension (TAC), Word Intelligibility by Picture Identification (WIPI). Central Institute for the Deaf Speech Intelligibility Evaluation (CID SPINE), Central Institute for the Deaf (CID), a set of sounds in the frequency range 300Hz-4kHz representative of the speech frequency spectrum, described by Daniel Ling, University of Western Ontario, Canada (LING sounds).

Online Resource 3 - Auditory outcomes data per included study

Article Title: Outcomes of cochlear implantation in Usher syndrome: a systematic review

Journal name: European Archives of Oto-Rhino-Laryngology and Head & Neck

Authors: HL Cornwall[1], CM Lam[1], D Chaudhry[2], J Muzaffar[3,4], P Monksfield[4], ML Bance[3,5]

Affiliations: [1] Cardiff and Vale University Health Board, Cardiff, UK. [2] College of Medical and Dental Sciences, University of Birmingham, Birmingham, UK. [3] Department of Clinical Neurosciences, University of Cambridge, Cambridge, UK. [4] Department of Otolaryngology, University Hospitals Birmingham NHS Foundation Trust, Birmingham, UK. [5] Department of Otolaryngology, Addenbrooke's Hospital, Cambridge University Hospitals NHS Foundation Trust, Cambridge, UK

Corresponding author: Professor Manohar L Bance, email: mlb59@cam.ac.uk
